# Strengthening the role of community health workers in supporting the recovery of ill, undernourished children post hospital discharge: qualitative insights from key stakeholders in Bangladesh and Kenya

**DOI:** 10.1186/s12913-021-07209-2

**Published:** 2021-11-15

**Authors:** Rita Wanjuki Njeru, Md. Fakhar Uddin, Scholastica Mutheu Zakayo, Gladys Sanga, Anderson Charo, Md. Aminul Islam, Md. Alamgir Hossain, Mary Kimani, Mercy Kadzo Mwadhi, Michael Ogutu, Mohammod Jobayer Chisti, Tahmeed Ahmed, Judd L. Walson, James A. Berkley, Caroline Jones, Sally Theobald, Kui Muraya, Haribondhu Sarma, Sassy Molyneux

**Affiliations:** 1 KEMRI-Wellcome Trust Research Programme, P.O. Box 230-80108, Kilifi, Kenya; 2 Nutrition and Clinical Services Division, icddr,b, GPO Box 128, Dhaka, 1000 Bangladesh; 3 Departments of Global Health, Medicine, Paediatrics and Epidemiology, University of Washington Seattle, Seattle, USA; 4 Centre for Tropical Medicine & Global Health, University of Oxford, Oxford, UK; 5 Department of International Public Health, Liverpool School of Tropical medicine, Liverpool, UK; 6 Research School of Population Health, The Australian National University, Canberra ACT, Canberra, 0200 Australia

**Keywords:** Community health workers, Undernutrition, Post-hospital discharge, Children, Acute illness

## Abstract

**Background:**

Undernourished children in low- and middle-income countries remain at elevated risk of death following hospital discharge, even when treated during hospitalisation using World Health Organisation recommended guidelines. The role of community health workers (CHWs) in supporting post-discharge recovery to improve outcomes has not been adequately explored.

**Methods:**

This paper draws on qualitative research conducted as part of the Childhood Acute Illnesses and Nutrition (CHAIN) Network in Bangladesh and Kenya. We interviewed family members of 64 acutely ill children admitted across four hospitals (a rural and urban hospital in each country). 27 children had severe wasting or kwashiorkor on admission. Family members were interviewed in their homes soon after discharge, and up to three further times over the following six to fourteen months. These data were supplemented by observations in facilities and homes, key informant interviews with CHWs and policy makers, and a review of relevant guidelines.

**Results:**

Guidelines suggest that CHWs could play a role in supporting recovery of undernourished children post-discharge, but the mechanisms to link CHWs into post-discharge support processes are not specified. Few families we interviewed reported any interactions with CHWs post-discharge, especially in Kenya, despite our data suggesting that opportunities for CHWs to assist families post-discharge include providing context sensitive information and education, identification of danger signs, and supporting linkages with community-based services and interventions. Although CHWs are generally present in communities, challenges they face in conducting their roles include unmanageable workloads, few incentives, lack of equipment and supplies and inadequate support from supervisors and some community members.

**Conclusion:**

A multi-pronged approach before or on discharge is needed to strengthen linkages between CHWs and children vulnerable to poor outcomes, supported by clear guidance. To encourage scale-ability and cost-effectiveness of interventions, the most vulnerable, high-risk children, should be targeted, including undernourished children. Intervention designs must also take into account existing health worker shortages and training levels, including for CHWs, and how any new tasks or personnel are incorporated into hospital and broader health system hierarchies and systems. Any such interventions will need to be evaluated in carefully designed studies, including tracking for unintended consequences.

**Supplementary Information:**

The online version contains supplementary material available at 10.1186/s12913-021-07209-2.

## Background

There has been a considerable decline in child mortality globally over the last two decades. However, mortality in vulnerable sub-groups remains a significant public health challenge in low- and middle-income countries (LMICs) [1]. Post hospital discharge mortality in acutely ill children often equals or even exceeds inpatient mortality [2, 3]. This risk is particularly high in undernourished young children, despite being treated using World Health Organisation (WHO) recommended guidelines during admission [4, 5].

According to the WHO guidelines for treatment of acute malnutrition, children admitted for inpatient management of complicated severe acute malnutrition should be discharged into Community Management of Acute Malnutrition (CMAM) programmes for continued monitoring and therapeutic support to recovery [6]. However, a review of the coverage of CMAM programmes in 21 countries shows that coverage is often sub-optimal [7]. Even where these programmes are in place, they require patients to be taken back to a facility, which is difficult for many families given distance and cost implications, and their additional domestic and income earning responsibilities [8, 9]. For these reasons, there is growing interest in the potential for Community Health Workers (CHWs) to offer home-based support for undernourished children [10].

Community Health Workers have been defined as “*lay members of the communities where they work, selected by the communities, answerable to the communities for their activities, supported by the health system but not necessarily a part of its organisation, and [as having] shorter training than professional workers*” (Lehman and Sanders 2007 p. 3) [11]. In some contexts, there are expanded definitions which include other community-based health providers, and some CHW programmes have been formally incorporated into health systems [12, 13]. Globally, there is renewed interest in the promotion of CHWs across most LMICs given healthcare access challenges for many people, and recognition that CHWs can build trust and rapport with patients and their families, foster therapeutic relationships and support development of plans of care that are acceptable to patients [14–17]. This is especially so following key global health directives – such as Universal Health Coverage and Leave No One Behind.

CHW programmes focus on a range of initiatives, especially health promotion activities [16–18] and have particular potential to support access and care in hard to reach rural and urban areas, and in areas experiencing acute shortages of formal health workers [14, 19]. In these contexts, they have been shown to contribute to reduced maternal and child morbidity and mortality and have supported referrals to health facilities [14, 20, 21]. Although several reviews have examined the role and functioning of CHWs in recent decades [13, 22], their specific role in supporting recovery of children post hospital discharge has not been well documented. Indeed, a recent literature and consultative review aimed at developing a research agenda for CHW programmes highlighted that “*there is need to test whether models of shared care involving referrals and counter referrals involving CHWs and health facilities can influence community health workers performance*” (Agarwal et al. 2019 p. 4) [23].

In this paper, we examine the actual and potential role of CHWs in supporting recovery of ill, undernourished children post hospital discharge in Bangladesh and Kenya. We draw on a review of policy documents, on interviews with family members of admitted children, CHWs and policy makers, and on observations in homes and hospitals.

## Methods

### Study setting

Undernutrition is still a major health concern in Bangladesh [24] and Kenya [25], and both countries have long-standing community health strategies that aim to bring health care closer to the people and enable them to take charge of their own health [26–28]. As in other settings, little is documented about the CHWs’ potential role in supporting recovery of admitted children post-discharge, including undernourished children.

#### Community health strategies

##### Bangladesh

In Bangladesh, community health system operate slightly differently in rural and urban areas. In rural Bangladesh, the Ministry of Health and Family Welfare oversees a community health strategy centred on a salaried and formally engaged and trained group of community-based health workers. These are the Community Health Care Providers based permanently at the lowest level of formal health facilities (community clinics); and Health Assistants and Family Welfare Assistants based at community clinics for 2 days a week and over the rest of the week in homes and outreach sites. In urban areas, the Ministry of Local Governments and Rural Development provides primary healthcare including to the urban poor through a contracted-out mechanism in which non-governmental organisations periodically bid for the services [29]. The service provider in Dhaka (the urban research site) at the time of writing was a United States Agency for International Development funded local non-governmental network of facilities known as *Surjer Hashi clinic* (smiling sun) where medical officers provide services, in addition to paramedics. CHWs referred to as Service Providers (SP) assist in the clinics and offer domiciliary services.

Across rural and urban areas in Bangladesh, there is also a wide network of CHWs programmes run by non-governmental organisations (NGOs). BRAC, a development NGO runs the largest CHW network (*Shasthya Shebika* (SS) and *Shasthya Kormis* (SK)), engaging approximately 46,000 CHWs across the country [30]. The SK are paid staff who supervise the SS volunteers, with each SS in charge of several households. SS volunteers conduct monthly visits and sell medical products provided by BRAC on credit, to enable them to earn an income.

##### Kenya

Kenya has had its Community Health Strategy in place since 2006, covering both rural and urban settings. The strategy was revised in 2014 following an evaluation in 2010 that highlighted shortcomings in management, remuneration and linkage mechanisms [27]. In 2010, Kenya also promulgated a new constitution that moved the country from a centralized system of governance to a devolved system. Following devolution and the subsequent formation of 47 semi-autonomous counties, community units as well as levels 2–4 of healthcare devolved to be the responsibility of county governments. The community unit is the first formal level of health care in Kenya. It consists of a population of 5000 persons, served by 10 volunteer CHWs known as community health volunteers (CHVs), supervised by five Community Health Extension Workers (CHEWs) and overseen by a Community Health Committee. This revision from the previous formation (which had one CHEW in charge of 50 CHVs) aimed to provide the CHWs with better technical support for service delivery. Each community unit is linked to a health facility, mostly a level two facility (a ‘dispensary’). A CHV is selected by and is supposed to be a well-trusted member of the community. He/she should serve the community they come from and work on a voluntary basis (albeit receiving incentives such as trainings and stipends for various activities). The CHEW on the other hand is a technical government employee with tertiary training in a health-related subject.

As in Bangladesh, a wide range of local and international NGOs work in the health sector in low income rural and urban areas in Kenya, including in urban informal settlements. Some NGOs employ their own CHWs while others engage the government volunteers for implementation of their often-vertical programmes such as on HIV, TB, nutrition and maternal and child health and offer financial incentives.

### The childhood illness and nutrition network

This paper draws on data collected as part of a social science sub-study conducted within The Childhood Acute Illness and Nutrition (CHAIN) Network’s cohort study. CHAIN is a multi-disciplinary research network aimed at understanding the mechanisms contributing to high mortality in acutely ill children during hospital admission and after discharge in LMICs despite care according to WHO and national guidelines, in order to gain a better understanding of the risk factors and mechanisms that could be targeted by interventions to reduce mortality [31]. The CHAIN cohort was conducted in nine hospitals in six countries across South Asia and Africa, including Bangladesh and Kenya. Each hospital recruited up to 500 acutely ill children aged below 24 months, stratified by nutritional status. The cohort had clinical and socioeconomic characteristics assessed at admission and discharge from hospital and had regular anthropometric assessments and biological samples collected during admission and post-discharge, including visits back to the study hospitals for scheduled follow-up at day 45, 90 and 180 post-discharge. Parents were reimbursed for transport and out of pocket expenses related to the follow-ups, and any illnesses identified were treated or referred to specialist services e.g. occupational therapy, cardiology.

### The qualitative sub-study in CHAIN

The embedded qualitative sub-study was conducted in 4 sites in Bangladesh and Kenya; a rural and urban site in each country [32–34]. The sites were purposively selected for their geographic, epidemiological and cultural diversity, and for their capacity to conduct in-depth qualitative work. Data collection differed slightly across sites and countries, in order to accommodate site specific interests and constraints.

#### The sub-study sites

In Bangladesh, the two hospital sites were International Centre for Diarrhoeal Disease Research (icddr,b) run hospitals: the urban Dhaka centre and the rural Matlab centre. These non-governmental hospitals provide free health care to all community members for diarrhoeal diseases, and treatment for respiratory illnesses for children, in addition to integrated maternal and child health services in the rural centre’s surveillance area. The rural Matlab facility hosts a health and demographic surveillance system where field staff conduct regular census and serve as CHWs in their assigned areas.

In Kenya, the two sites are government hospitals: one in Kilifi, a rural low-income coastal town and one in the capital city Nairobi, whose catchment area includes a nearby large informal settlement (colloquially referred to as ‘slums’). Both are county referral hospitals or ‘level 4’ hospitals providing both outpatient and inpatient services, as well as some specialist services. CHAIN supported adherence to national treatment guidelines in both countries. More detailed descriptions of the communities in the catchment areas of these hospital sites are elsewhere [32–34].

#### Data collection

We conducted repeat in-depth interviews with family members of 64 CHAIN cohort children from across the 4 sites: 42 in Kenya (20 rural and 22 urban); and 22 in Bangladesh (11 rural and 11 urban) selected during admission. Children were purposively selected to have diverse biological and socio-economic vulnerabilities (based on nutrition status and level of social disruption); 27 children from across the four study sites had been categorised by CHAIN as having Severe Wasting on the basis of mid-upper arm circumference (MUAC) measurement, or Kwashiorkor (SWK). The rest of the children had moderate wasting (MW) (also by MUAC) or no wasting (NW). MUAC was selected by CHAIN as the most appropriate measure of nutritional status among very sick children at the time of admission to hospital. Summary characteristics of the children and households are presented elsewhere [32–34], but all children were aged 2–23 months, and most had experienced a social disruption within a few months prior to admission (such as carer’s illness, movement from one area to another, birth of a sibling, death of a parent or loss of family income).

We held interviews with children’s family members in their homes following their discharge from hospital, with the first interview within a week of discharge, and up to three further visits over the following six to 14 months. Interviews exploring their perspectives on their child’s illness and associated treatment-seeking journeys. In later visits, we asked specific questions about interactions with CHWs pre-, during and post hospital discharge; and if and how CHWs assisted them in their treatment-seeking pathways. In visiting children’s home up to four times over several months, sometimes staying in the homes for hours, we observed families’ situations and witnessed first-hand some of the struggles they faced; particularly family members of children with SWK. The interviewed carers were mostly mothers, and/or other family members such as child’s grandmother, aunts, and in few cases, fathers.

To examine the *potential* role of CHWs in post-discharge care, we specifically asked family members in all 42 households in Kenya their perspectives regarding the opportunities for CHWs’ involvement for families like them. We did not have the equivalent data in Bangladesh, and so interviewed 11 key informants to gather their views: seven CHWs from the range of institutions working with CHWs across the country; and four senior staff working with CHWs in the Ministry of Health, NGO and research sectors. These interviews were held in places convenient to participants, including clinics and offices. All interviews with carers were conducted in their preferred language (Kiswahili, Kiswahili or Giriama and Bangla in Nairobi, Kilifi and Bangladesh respectively). Interviews with CHWs were conducted in Bangla and those with senior officials conducted in English. Two and occasionally three study team members were present for each interview to conduct the interview, take notes and observe, with interviews taking 50–100 min. CHW questions were generally embedded within broader semi-structured interview guides (see CHW elements in supplementary file 1).

We supplemented the above interviews and observations with a document review to understand the policy guidance for post-discharge care and the involvement of CHWs in both countries. Government guideline documents of management of child undernutrition and related community health strategies were reviewed. Attention was paid to the post-discharge period, but it was necessary to understand the whole pathway to identify where the chain breaks. The review focused on documents in English. Although our study design does not support conclusive statements of data saturation, we are confident on the main themes of our findings because they arose across multiple data sources and methodological approaches.

#### Data management and analysis

All interviews were audio recorded, transcribed, translated (where necessary) and managed using NVivo software. Summaries and household narratives were developed as described elsewhere [33], and detailed findings on families’ health seeking) [33] and gendered decision making for health seeking for children in Kenya [34] and Bangladesh [32].

For this paper, we drew on the household summaries and narratives that documented family experiences throughout their child’s illness. We also conducted additional analyses utilising the framework approach, combining deductive themes based on the research questions, and inductive themes emerging from re-reading of transcripts and summaries. Initial coding was done in NVivo by team members- who had primarily collected the data- from the various sites (rural Kenya- RN, SZ, GS, AC, MK; Urban Kenya-KM, MO, MM; Bangladesh-MFU, AI, AH). Data collectors included both men and women, all either from the local area with knowledge of the first languages or from the same country. Charts were developed to condense the data and support comparison. For the household data, this entailed exploring knowledge of and interactions with CHWs, and the perceived potential of CHWs to contribute to children’s recovery. For the key informants’ data, the charts were used to organise perceptions about current and potential CHW involvement in post-discharge care, including barriers and facilitators. RWN developed the charts with input from MFU and SM.

The authors of this paper were primarily social scientists, clinicians and clinical researchers working in various roles in the CHAIN study and involved in one or several of the sites. Indeed, the first ten and the last three authors were members of the social science team. Involved also were external expertise in CHWs (ST) and in service delivery (CJ).

#### Ethical approval

The cohort study received ethics approval from the Kenya Medical Research Institute Scientific and Ethics Review Unit (KEMRI/SERU/3318/054), the Oxford Tropical Research Ethics Committee (OxTREC number 34–16) and the icddr,b Ethics Review Committee (CHAIN Social Science Sub-Study PR-16056). The cohort study obtained written parental consent for the children’s participation. The data used in this paper involved interviews with carers, community health workers and key informants. Administrative clearance was obtained from the various heads of health departments in the respective study sites and written, continuous, informed consent obtained from all participants. Where there were repeat encounters, verbal consent was sought for continued participation.

## Results

We begin by highlighting what guidelines for care for undernourished children say CHWs can/should do. We then lay out family members’ actual experience with CHWs and the potential role of CHWs to better support undernourished children post-discharge. Next, we highlight some of the immediate influences on the lack of use of CHWs in practice, including the CHAIN cohort study itself, communication between levels of care, and families’ perceptions and knowledge of the role of CHWs. Finally, we briefly describe the broader influences on CHW functioning and reputation such as training, remuneration and supplies and equipment as well as workload issues.

### The role of CHWs in supporting undernourished children post-discharge – what should happen

We reviewed two main sets of guideline documents concerned with how services should be delivered to undernourished children and how community level health services involving CHWs should be run: guidelines for the management of acute malnutrition and community health strategy documents respectively.

Regarding inpatient and outpatient care for undernourished children, in Bangladesh there were two sets of national guidelines; one for the facility-based management of children with severe acute malnutrition, and another for the community-based management of acute malnutrition (CMAM) [35, 36]. In Kenya, the Integrated Management of Acute Malnutrition (IMAM) guides both inpatient and community-based management of children with undernutrition [37]. In both countries, these documents are based on World Health Organisation guidelines. Key relevant elements are highlighted in Table 1. In brief, children with complicated severe acute malnutrition should be managed as inpatients and upon resolution of the complication, be discharged into the community/outpatient programme where uncomplicated severe acute malnutrition and moderate acute malnutrition are managed. CMAM in both settings is supposed to be facility but outpatient-based, with CHWs ideally playing a screening and referral as well as follow-up role at the community level. In both countries, the specific mechanisms to support links between hospital admission/discharge processes and CHWs are unclear. In Bangladesh, guidelines suggest the use of a transfer slip, and in Kenya a conversation between a health worker and CHW is implied. Moreover, in Bangladesh, there is no nationalised CMAM implementation.

**Table 1:** Summarising guidelines in Bangladesh and Kenya

	Inpatient and CMAM Bangladesh	IMAM Kenya	CHW strategy Bangladesh	CHW strategy Kenya
**Admission**	Management of Complicated severe acute malnutrition is inpatient		Acknowledges role of CHWs in malnutrition, and in referral to hospital	No acknowledgment of role of CHW Acknowledges poor linkage between community and hospital levels
**Discharge criteria**	Upon resolution of complication and to be managed according to CMAM guidelines		N/A	
**Linkage to support at discharge**	Transfer slip with description of monitoring plan for specific patient	CHW should discuss inpatient child’s requirements with health worker to understand post-discharge health needs	No specific reference to referral back to CHWs from hospital	
Post-discharge support	On discharge children should be sent for follow-up to the nearest public or NGO health facility and followed-up regularly, also enrolled into CMAM, included in growth monitoring sessions at health centres, and enrolled into safety net programmes According to CMAM: Management in the community health facilities with some patients followed-up at home in case of default or “other challenges”. CHWs can provide home-based care for children with SAM without referral in the absence of any complications.	CHW should discuss with the child’s carer the appropriate home environment to facilitate recovery; and ensure CHW is contactable if there are any concerns about the child’s recovery “well trained and motivated” CHWs should work at the community-based health facility to monitor children on treatment and assist in the distribution of nutrition therapeutic products.	Follow-up of patients with chronic conditions eg TB. Malnutrition not mentioned at all	Malnutrition not mentioned specifically at all in the guidelines

The community health strategies for both countries [27, 28] detail the specific roles of CHWs as rehabilitation, and follow-up of patients with chronic illnesses; with the importance of CHWs’ capacity building highlighted. However there is little specific information on the role of CHWs during hospital admission, on discharge and post-discharge. In Kenya, the CHW guidelines do not mention malnutrition in CHW roles.

Table 1 provides a summary of what the guidelines say *should* happen.

### The actual and potential role of CHWs in supporting undernourished children post-discharge in practice – the case of four CHAIN sites

Drawing on both spontaneous and prompted responses (with prompted information from 58 of the 64 households we visited; four children had died, one carer in rural Kenya site refused without explanation and one in urban Kenya was unreachable), few interactions with CHWs were reported across the entire treatment-seeking pathway for admitted children, even for those discharged after complicated Severe Wasting or Kwashiorkor (SWK).

In Kenya, no family mentioned being referred to CHWs post-discharge, and only two rural families, both with children categorised as SWK at admission, reported having interacted with a CHW at all for the illness episode. In the first case, it was before the onset of the malnutrition and only a brief encounter. In the second case, the child’s grandmother reported that a CHW referred her to a health facility when the child was unwell, prior to admission, by making a phone call ahead to the hospital. The grandmother was however upset because when she got to the hospital, she was not promptly attended to as expected, and instead spent the whole day queuing with everybody else. She mentioned that she would have preferred that the CHW accompanied her to the hospital. Moreover, she complained that when the child was not admitted, CHWs visited the home to show each other the child’s symptoms and discuss why the child had not been admitted. When the child was later admitted and then discharged, one CHW upset the grandmother by expressing shock that the child had survived. Only one urban family reported direct interactions with a CHW; in this case being phoned by an NGO-based CHW after discharge and asked about the child’s health. This CHW had offered to visit the home but this had yet to happen.

In Bangladesh, only one family reported having interacted with a CHW across the treatment-seeking pathway, in this case post-discharge. However, the CHW’s visit was a prenatal care visit and they did not enquire about the discharged child’s health, despite the child having been admitted with SWK. Although another two families in Bangladesh interacted with CHWs at the icddr,b community health facility after discharge, they were not visited at home regarding the child’s illness.

To learn more about the perceived potential role of CHWs, we asked family members in Kenya and CHWs in Bangladesh their views on this. Family members of just under half of all the children (and just over half of those with SWK) in Kenya felt that more involvement with CHWs would assist families facing similar challenges to theirs, including through door-to-door visits to help family members identify danger signs, advice, and referring to health facilities where necessary. Several families also noted that CHWs could help families reduce health-related costs by preventing them from making unnecessary visits to hospital which in turn cost money, or – when necessary – by accompanying them to health facilities to support access to prompt care. One mother explained how a CHW might have assisted her:

*“Yes, they [CHWs] could have helped me that time. Maybe they could have seen the child has deteriorated and she is not feeling well. I think they could have helped me either by giving me medication before I went there [health facility] or if they don’t have medication then they could have taken me to the hospital. I wouldn’t have had the trouble of going here and there first, since they will be with [you] … they could have helped me because when we’d come there [health facility] together, they could have seen doctors and told them, “ I have brought a patient here , he/she is suffering from this and this” so you get help fast”.* (Mother, Household 017, SWK, Kenya Rural)

In Bangladesh, CHWs mentioned they could assist in similar areas cited in Kenya, and with nutrition advice post-discharge. As one CHW explained:

“*it is obviously important to come to [CHWs] after discharge period to ensure follow-up counselling because during admission or when patients go to the Upazila [sub-district] level hospital to receive treatment it is not always possible to get counselling from the hospital physicians or nurses or health workers because of patient loads*”. (Male, Community Health Care Provider, Bangladesh, rural)

CHWs in Bangladesh also felt that CHWs placed in hospitals (for example those funded by BRAC and based in public hospitals to help patients navigate the facilities and avoid ‘brokers^1^’) could create linkage of patients back to (community-based) CHWs after discharge, for careful monitoring and support.

That there is some potential for CHWs to help families was supported by our observations in households, our broader learning about influences on treatment-seeking pathways, and the questions we were asked during the qualitative work. For example, regarding follow-up care and advice for home-based care for recovering children, some carers did not remember what information they were given at discharge or found the advice hard to follow due to financial constraints or unavailability of recommended food items in their localities.

In some cases, especially in the Kenyan urban site, mothers chose to quit their jobs to stay at home and focus on supporting the child’s recovery. However, in other cases, childcare post-discharge was at least partially handed on from the person who was with the child during hospital admission (usually the mother) to others in the household (relatives and elders in the home, usually female) who had not directly received health care messages. Information was therefore often lost, or – given age and gender power relations in homes – not valued. In Bangladesh, one caregiver therefore mentioned that it would be helpful to have a respected outsider (such as a CHW) to communicate with her husband and other elder family members.

Furthermore, in several cases we referred family members back to the CHAIN clinicians for follow-up care or advice (beyond what was required as part of the study follow-up) and referred them to additional sources of support such as food distribution NGOs, or gender-based violence groups; to ease broader influences on families’ vulnerability. These are all potential roles that CHWs could take on.

### Factors contributing to low use of CHWs among our participants post-discharge

#### CHAIN study design and functioning

An important potential factor influencing the low use of CHWs (*post-discharge*) in our study sites may have been the study design itself. As outlined in our protocol [31], the CHAIN cohort had regular anthropometry measurements taken, and samples collected during and post-discharge, including visits back to the study hospital for follow-up at day 45, 90 and 180 post-discharge. Parents were reimbursed for transport and out-of-pocket expenses incurred during the study processes, and any illnesses identified during these visits were treated or referred to specialists.

Families across all sites hugely appreciated these follow-up activities. As we have described elsewhere [38], some families in Kenya were even disappointed that the follow-ups had ended because they would no longer have their children regularly checked. Others were relieved that the follow-up visits had ended because they felt it meant that their child was no longer ill. This shows the perceived (and real) overlap between research related cohort follow-ups and clinical care, and highlights that support is valued by many families after hospital discharge. In addition to the study specific follow-up activities, in Kenya, family members reported being advised to go to the nearest health facility for any health care needs that emerged post-discharge. In Bangladesh, carers reported being given a hospital contact person and number to call if they experienced any problem with the child’s health. These links to physicians, together with the follow-up clinics, may in some ways have replaced any need they may otherwise have had to contact a community-based facility or CHW. As one urban Bangladeshi parent put it:

“*I never went to the health workers at the community health facility because they are not as educated as physicians. Also, I don’t feel like going there because I can easily take suggestions from physicians of icddr,b over mobile phone. So I don’t think I need to communicate with any CHWs to seek treatment advice for my child’s illness*” (Mother HH62 MW, Bangladesh urban).

The above quote also suggests an appreciation among families of the higher training of clinicians over CHWs, discussed more below. However, five families did not contact icddr,b physicians: they were concerned this might result in their being asked to bring their children to the hospital, which was inconvenient and costly (in time and transport). Two children therefore received treatment from a nearby facility and drug shop instead. This further suggests that closer to home monitoring would be useful.

#### Communication between levels of health care

We noted that there was rarely any direct communication reported between the CHAIN staff and CHWs across the four Bangladesh and Kenya sites, possibly as a result of the lack of clarity on how to do this in guidance, as well as the follow-ups built into CHAIN (described above). The one exception was in the rural Bangladesh site, given the hospital links with the health and demographic surveillance system. In our interviews with the rural community based icddr,b employed CHW, she reported occasionally being notified when a particularly vulnerable child had been discharged, especially when the CHW had themselves referred the patient to the hospital. In a general comment, not specific to children with undernutrition, the CHW commented:

“*One mother from my community came back home from our icddr,b hospital with her child without involving the hospital physicians. The hospital authorities got in touch with me over the mobile phone and told me that ‘this mother went home too early, please motivate her to come back again to the icddr,b hospital to take treatment as per medical guidelines’. In that case I went to the home of the mother and motivated her to take treatment as per instructions of the physicians*” (Female icddr,b, Community Health Research Worker, rural Bangladesh)

Without specific links to inpatient management of undernutrition, more generally in communities, including for public hospitals, CHWs in Bangladesh reported that they only tend to find out about discharged vulnerable patients serendipitously when they go door to door for their regular visits, when they make a specific effort to keep in touch with patients that they referred to the hospital, or when parents contact them after discharge. According to CHWs and key informants, the lack of formal communication between hospitals and CHWs post-discharge is compounded by challenges with CHW supervision and lack of respect among health workers for CHWs:

“*Sometimes the seniors or the paramedics they don’t care much about the community health workers and even sometimes the doctors they don’t show much care. In this area there are some gaps*” (KII 003 Bangladesh).

#### Families’ knowledge of and perceptions about CHWs and their roles

From our interviews with household members across both settings, it was clear that another important influence on non-use of CHWs was likely to be their own familiarity with CHWs, and their views on the ability of CHWs to assist them.

Regarding *familiarity with CHWs*, in Kenya, eight of the 40-households members asked reported never having heard about CHWs in the community and an additional eight reported having heard about but never interacting with them. Although we do not have corresponding figures for Bangladesh (see methods), there appeared to be greater familiarity with CHWs among families in Bangladesh. Nevertheless, in Bangladesh three of the 11 urban families reported that they had not interacted with CHWs post-discharge as they had only recently moved to the area and did not know them or their way around.

“*I do not know any CHW of this area since I moved here from another urban slum in the city recently. After discharge no CHW visited my household. I don’t know whether there are any services in this area to visit children after discharge to provide services. That’s why when my child suffers from illness, I seek treatment from the pharmacy or local drug shop*.” (Mother, Household 052 SWK, Bangladesh, urban)

Regarding *families’ perceptions of CHWs’ ability to assist post-discharge*, there were clearly concerns among family members in both countries about their potential value. In Bangladesh, some participants did not know if CHWs could help or felt that CHWs do not have the capacity to handle their child’s illness. Also noted by families in Bangladesh is that CHWs were often absent from community clinics. In Kenya, most CHWs were associated with intermittent externally funded activities in communities (such as deworming, mass immunization campaigns and bed nets distribution) rather than with post-discharge care.

Regarding CHWs’ *potential ability to assist post-discharge*, socioeconomic status and gender appeared to play a role in their acceptability in communities. For example, CHWs themselves in Bangladesh sometimes felt that community members did not respect their advice, especially when they were from a lower socioeconomic status than the family members they were advising. Regarding gender, in Kenya, carers felt that a female who is a mother herself would be best placed to support after discharge. In Bangladesh, although women generally appreciated counselling from female CHWs, they reported that this advice was sometimes less valued by male household members. Additionally, in Bangladesh, CHWs are recognised in some areas to have a role in challenging domestic violence in households. This role can upset men in some homes, with potential implications for post-discharge support for children [32].

### Broader influences on the potential for CHWs to support post-discharge care

Beyond the above issues, our respondents highlighted that there are broader challenges that would need to be overcome for CHWs to play a stronger role in supporting families with undernourished children post hospital discharge. Issues they raised included adequate training, provision of required equipment and supplies, and motivation. Regarding training, stakeholders highlighted that CHWs would need specific training in undernutrition in children, in nutritional needs and how these can be met using cheap, locally available supplies; and in when and how children need to be referred back to facilities. They also highlighted the need for relevant information, education and communication materials to support interactions with mothers and other family members, as well as – depending on their precise roles - weighing scales, nutritional products, and phones (or at least airtime) and contact numbers to facilitate referral to health facilities and other community-based support services.

CHWs felt that training, equipment and supplies can support motivation, including through strengthening their confidence and interactions with family members. However, key informants highlighted that overall workload, as well as supervision and support processes, are also important; that any initiatives would need to consider how any new roles would relate to roles CHWs already have, as well as supervision and support processes to deal with conflicts. They also noted that whether or not there would be any form of financial motivation needed careful consideration. In Bangladesh, CHWs reported that per visit remuneration was motivating in visiting pregnant women, as it supports them in ‘*running to identify the pregnant women’*, but many CHWs also indicated a more intrinsic motivation:

“*the thing that inspires me most for example is if a mother says my family is doing well, we are living a good life, because of prevention of illness from your counselling, from this perspective the mother says that we are leading a good life because of you, this inspires me to work more*” (Female, BRAC SS, urban)

## Discussion

To consider the potential for community health workers (CHWs) to support the recovery of ill, undernourished children post hospital discharge in Kenya and Bangladesh, we reviewed the relevant national guidance, and interviewed family members of children who had been admitted with acute illnesses in hospitals, as well as other key informants. Here, we discuss three key findings in turn: the guidelines on involvement of CHWs in post-discharge care for undernourished children compared to findings and our observations of practice; the potential lessons from the CHAIN design for CHW interventions; and the wider influences on CHW roles and functioning.

In both countries, guidelines for care for undernourished children include post-discharge care involving the facility-based community management of acute malnutrition (CMAM) that utilises primary health care systems. However, the specific mechanisms to support links between hospital admission/discharge processes and CHWs are unclear and in Bangladesh CMAM has not been implemented nationally. In practice, post-discharge referral to primary care facilities and especially CHWs was infrequent and appeared largely passive, with some families making their own links with CHWs, and some CHWs reaching out themselves to families. As noted by Wiens and colleagues [9], such passive referral approaches are problematic: parents prefer to avoid further facility visits due to time and financial costs, and parents are under pressure to fulfil responsibilities neglected during hospitalisation. These challenges were experienced by many households we interviewed, and especially by families whose children had lengthy hospital stays, as described in greater depth elsewhere [32–34]. Lengthy hospital stays are common for severely wasted children in many settings [39] and in our study were most common for children classified as SWK and diagnosed with other chronic illnesses [32–34].

Nemetchek et al. [3], drawing on a systematic review across LMICs have argued that post-discharge interventions in general are more likely to work if implemented before a child leaves the hospital. Wiens et al. [9] in their Ugandan study examined the effectiveness of introducing a bundle of interventions on discharge, including a referral form to a facility or CHW along with a brief educational counselling session and a discharge kit. Their study, which compared outcomes with a historical cohort, suggested that a formal process on discharge to facilitate links with CHWs, overseen by a focal person such as a discharge nurse, led to greater use of CHWs post-discharge, and greater levels of hospital readmission [9]. The authors note that *‘... post-discharge care should be an important component in the care of all discharged children, [but that] limited resources and an already strained health system are likely to be major barriers in its implementation.’* (Wiens et al. 2016 p. 432) [9]. To encourage scale-ability and cost-effectiveness of such interventions, they argue that there should be an identification of the most vulnerable, high risk children on discharge [9]. Our findings support the potential importance of a systematic approach on discharge to post-discharge care, including clarity in guidelines on what is available for different vulnerable groups, how CHWs should be contacted, and who should oversee the process using what paperwork. Any such approach, however, must also recognise existing training levels and the shortage of both hospital and community-based health workers. Undernourished children, especially those diagnosed with severe malnutrition and other chronic illnesses, should be included in guidance and practice as potentially high-risk groups needing special consideration in any targeting.

Notable in our work was that CHWs rarely featured spontaneously in family members’ stories. This absence of CHWs in the post-discharge period may at least in part be explained by the CHAIN study design where CHAIN staff took on some possible CHW roles for the several years the study was conducted. These CHAIN activities, and the wider literature, suggest potential activities and approaches to strengthen CHWs’ roles post-discharge. Firstly, CHWs could provide a communication link for families between hospital staff and primary health care facilities, including through being reachable by phone, and being able to make phone contact themselves with facility staff; an approach supported by the high proportion of households who now own or have access to mobiles in LMICs [40], and the growing number of phone-based health interventions [41]. Secondly, CHWs could support with the implementation of cash transfers to ensure that funds are available to assist households with accessing a hospital or health centre for follow-up care where needed. A study in the Congo found that unconditional cash transfers to families decreased the proportion of relapse to SAM following treatment [42] and O’Sullevan et al [43]- drawing on that study – have argued that determining whether such an approach can work in other settings is a priority. We suggest that future studies should consider the potential role of CHWs in such interventions, for example in providing information and reminders to households about accessing funds, or – possibly more challenging – assisting in administering them (for example through clarifying household eligibility or following up on use of transfers).

Even in the context of the CHAIN follow-ups being in place, we identified opportunities for CHWs to assist families post-discharge, including regarding provision of context sensitive information, identification of danger signs and supporting with referral. This is in addition to being a trusted outsider in the contexts of mothers’ constrained power in relation to husbands and elder female relatives [34, 44]. Studies have demonstrated that CHWs can assist families with information that leads to more prompt health seeking and reduced harmful practices, including through challenging beliefs that some symptoms – such as diarrhoea or weight loss when teething – are normal [33]. Two studies from western Kenya for example - one on malaria treatment [45] and the other on chronic diseases management (HIV, hypertension and TB) [46] - reported that CHWs were generally positively perceived by patients, caregivers and community members, and that this relationship contributed to their use for advice and referral, and to their positive impact. These studies also suggested that CHWs were able to assist families reduce treatment-seeking costs through reducing unnecessary facility visits. Furthermore, for undernutrition specifically, recent intervention studies in Mali and Uganda suggest there is significant potential for CHWs to assist with nutrition care closer to the community and to improve post-discharge outcomes [8, 9]. Our findings support the potential for CHWs to assist families post-discharge, including those with undernourished children.

Despite the potential for CHWs to play a positive role in post-discharge support, there are a range of possible challenges. Families having concerns about CHWs’ competence to perform some of their roles and about their trustworthiness (ability to “keep secrets”), and conflicting information between CHWs and hospitals, have been documented as challenges in many LMIC settings, including Kenya and Bangladesh [45, 47–49]. CHWs need adequate training and provision of supplies to perform what can be multiple and complex roles [13], to promote acceptability and respect among communities and to build positive relationships with health workers and managers [48]. Moreover, integration of any new roles within existing roles and appropriate supportive supervision and monitoring is essential [13, 50], including to address challenges of over-verticalisation of care (siloed interventions that can lead to missed opportunities to offer support to vulnerable cases) [51–53]. The latter was observed in our study where CHWs’ visit to a home focused on antenatal services, missing an opportunity to follow-up on the recovering child’s condition. This may be linked to which services are incentivised, because previous studies have argued that incentives promote home visits by CHWs [54].

Our study was limited in terms of depth of exploration of factors influencing CHW potential and functioning, because specific questions were only included in the final encounters with families, and we were unable to seek CHWs perspectives from the Kenyan sites specifically on their actual and potential role because the covid-19 pandemic curtailed fieldwork. Although this has implications for our ability to make concrete comparisons across sites, the paper lays the groundwork for specifically designed in-depth studies across a range of rural and urban settings. Further research planned in the Kenyan urban site will examine the implementation of the community health strategy. In Bangladesh, further research is needed in areas served exclusively by government CHWs. Despite these limitations, we are confident that the insights from family experiences, key informants and informal observations shared in this paper can feed into more specifically designed implementation research to support vulnerable children post-discharge. Any such interventions must recognise and ideally work to transform the deeper structural determinants of child and household well-being such as widespread poverty, gender inequity and low access to affordable quality health care. This is particularly important in the context of the COVID-19 global pandemic, which is exacerbating hunger and inequity [55] with context sensitive protections and responses being particularly needed [56]. CHWs have an important role in such initiatives, but require appropriate support and protection [57].

## Conclusion

We identified many opportunities for CHWs to assist families post-discharge, including through providing context sensitive information and education, identification of danger signs, supporting with linkages between different health providers and other essential services, and supporting with the implementation of targeted interventions such as cash transfer programmes.

We support others in recommending the introduction of a systematic multi-pronged approach before or on discharge, supported by clarity in context-specific guidelines on what is involved for different vulnerable groups, how CHWs should be contacted, and who should oversee the process using what paperwork. To encourage scale-ability and cost-effectiveness of any such interventions, there should be a targeting of the most vulnerable, high-risk children, including undernourished children. Intervention designs must also take into account existing health worker shortages and training levels, including those faced by CHWs. Awareness creation among communities on the presence and role of community health workers is needed, as well as strategies to address broader structural challenges that contribute to undernourished children’s vulnerability to mortality. Any such interventions will need to be evaluated in carefully designed studies.

## Supplementary Information

**Additional file 1.** Interview guides- CHW elements. Contains the selected question from the interview guides used for household and key informant interviews from the broader tools used for the study.

## Data Availability

The datasets used and/or analysed during the current study are available from the corresponding authors on reasonable request.

## Abbreviations

CHAIN: Childhood Acute Illness and Nutrition Network
CHEW: Community Health Extension Worker
CHV: Community Health Volunteer
CHW: Community Health Workers
CMAM: Community Management of Acute Malnutrition
HH: Household
HIV: Human Immunodeficiency Virus
icddr,b: International Centre for Diarrhoeal Disease Research, Bangladesh
IMAM: Integrated Management of Acute Malnutrition
KII: Key Informant Interview
LMICs: Low and Middle-Income Countries
MUAC: Mid Upper Arm Circumference
MW: Moderate Wasting
NGO: Non-Governmental Organizations
NW: Not wasting
SAM: Severe acute Malnutrition
SK: Shasthya Kormis
SS: Shasthya Shebika
SWK: Severe Wasting or Kwashiorkor
TB: Tuberculosis
WHO: World Health Organisation

## Footnotes

1 Brokers in Bangladesh include individuals in and around government hospitals such as health workers, support staff and even rickshaw pullers who are recruited by private health facilities to direct patients there-with a promise of better and more prompt care and are paid a commission.
