# Supplementary material for: Strengthening the role of community health workers in supporting the recovery of ill, undernourished children post hospital discharge: qualitative insights from key stakeholders in Bangladesh and Kenya
Source: BMC Health Serv Res. 2021 Nov 15;21:1234. doi: 10.1186/s12913-021-07209-2 (PMC8590969; doi:10.1186/s12913-021-07209-2)
Supplement: Supplementary file 1 — Additional file 1. Interview guides- CHW elements. Contains the selected question from the interview guides used for household and key informant interviews from the broader tools used for the study. [file 12913_2021_7209_MOESM1_ESM.docx]

**Supplementary file 1: Interview guides- CHW elements**

**Household interviews:**

**Within visit 2**

(Following detailed overview of treatment-seeking journey and information on each action)

Have you had any support from others in the community in managing your child’s illness and its effects on your family?

- Anyone in the community?
- What about community health workers – are there many living and working in this area? Do you know them personally? Have you interacted with them? Are they helpful? In what way? What makes you say that?

**Within visit 3**

We have discussed in previous interviews about **community health workers**. [explain what a CHW is]. You mentioned you did[n’t] know any in your area/local facility. Is that right? Have you come across any more recently? Tell me about that….

- Were you referred to a CHW at any point in your child’s illness?
- What is the potential do you think of CHWs to support families like yours in the case of illness? What would you most need from them? At what point of the illness/recovery?
- What would families be most keen on in such a person? What would they most worry about? Eg Re where they are from? Whether or not they are linked to a hospital? Male or female?

**Stakeholder interviews**

- For the children managed in-patient: what **should happen at discharge** in terms of information given and **referral.**

**Probes include:**

- - Do CHWs play a role in any of these processes? What are these roles?
  - How does the referral process work? Is the link direct with homes or through peripheral facilities or CHWs?
  - How if at all does communication with other health facilities or with CHWs happen?
- **Tell me about any other involvement of community health workers in hospital activities**
  - – eg discharge for other patients? eg HIV, TB other NCDs)
  - eg other activities in the hospital (probes - whether disease focused, receiving patients and referrals, communication with other facilities about the patients)
  - eg other follow-up activities post discharge from the hospital?
- **How would the linkage from the hospital to the community best happen?**
  - What do you think would be needed by CHWs from facilities? From families?
  - Challenges envisioned (probe support, resources and workload, supervision and performance monitoring?)
  - Additional training needed?
  - Use of mobile phones: what role does/can mobile phone technologies play
